# Comprehensive discovery of DNA motifs in 349 human cells and tissues reveals new features of motifs

**DOI:** 10.1093/nar/gku1261

**Published:** 2014-12-10

**Authors:** Yiyu Zheng, Xiaoman Li, Haiyan Hu

**Affiliations:** 1 Department of Electrical Engineering and Computer Science, University of Central Florida, Orlando, FL 32816, USA; 2 Burnett School of Biomedical Science, College of Medicine, University of Central Florida, Orlando, FL 32816, USA

## Abstract

Comprehensive motif discovery under experimental conditions is critical for the global understanding of gene regulation. To generate a nearly complete list of human DNA motifs under given conditions, we employed a novel approach to *de novo* discover significant co-occurring DNA motifs in 349 human DNase I hypersensitive site datasets. We predicted 845 to 1325 motifs in each dataset, for a total of 2684 non-redundant motifs. These 2684 motifs contained 54.02 to 75.95% of the known motifs in seven large collections including TRANSFAC. In each dataset, we also discovered 43 663 to 2 013 288 motif modules, groups of motifs with their binding sites co-occurring in a significant number of short DNA regions. Compared with known interacting transcription factors in eight resources, the predicted motif modules on average included 84.23% of known interacting motifs. We further showed new features of the predicted motifs, such as motifs enriched in proximal regions rarely overlapped with motifs enriched in distal regions, motifs enriched in 5′ distal regions were often enriched in 3′ distal regions, etc. Finally, we observed that the 2684 predicted motifs classified the cell or tissue types of the datasets with an accuracy of 81.29%. The resources generated in this study are available at http://server.cs.ucf.edu/predrem/.

## INTRODUCTION

The comprehensive discovery of DNA motifs is fundamental to the global understanding of gene regulation. DNA motifs, often represented as consensus sequences or position weight matrices, are common DNA sequence patterns bound by regulatory proteins (1). One major type of regulatory proteins is transcription factors (TFs). Several hundred TFs may be active under an experimental condition (2). Multiple active TFs often bind short DNA regions of several hundred base pairs (bps) called cis-regulatory modules (CRMs) to control the temporal and spatial expression patterns of target genes (3). It is thus essential to identify motifs of all active TFs under an experimental condition to gain a global view of gene regulation under this condition.

The advent of several next generation sequencing based biotechnologies provides an unprecedented opportunity to discover DNA motifs of active TFs. TF-based chromatin immunoprecipitation followed by massive parallel sequencing (ChIP-seq) experiments can pinpoint potential binding regions of one TF under a given condition (4,5). These regions, each on average several hundred bps long, can aid the comprehensive discovery of cofactor motifs of the TF under consideration (6). Histone-based ChIP-seq experiments may indicate all potential enhancers and promoters bound by TFs under an experimental condition (7–9). These regions, each often several thousand bps long, may contain binding sites of all active TFs. Finally, DNase I hypersensitive analysis followed by sequencing (DNase-seq) defines the DNase I hypersensitive sites (DHSs) that are accessible for TF binding (10). Because DHSs are only several hundred bps long and likely contain the majority of TF binding sites (TFBSs), DHSs are viable for the comprehensive discovery of DNA motifs. For instance, a recent study showed that 98.5% of TFBSs mapped by TF-based ChIP-seq experiments in the Encyclopedia of DNA Elements (ENCODE) project are located in DHSs defined by DNase-seq (11).

Many computational methods are available for motif discovery. Most existing methods (12,13) are designed for small instead of large datasets. A handful of more recently developed methods (14–20) identified *bona fide* motifs in TF-based ChIP-seq datasets. Despite the existence of these methods, to our knowledge, few studies have attempted to comprehensively *de novo* discover motifs in histone-based TF binding regions or DHSs under a given experimental condition.

Recently, a new method, SIOMICS, successfully identified most cofactor motifs in 13 TF-based ChIP-seq datasets and no motif in 13 random datasets (6). Because the initial version of SIOMICS (6) considered only motifs of a fixed length, which is not the case in practice, we further extended it to predict motifs of variable lengths and showed that the extended SIOMICS had higher accuracy in motif discovery in the aforementioned 26 datasets (21). For simplicity, SIOMICS below refers to the extended SIOMICS. Compared with other motif discovery methods developed for ChIP-seq data analysis, SIOMICS has two special features that make it a promising tool for comprehensive motif discovery: (i) SIOMICS considers the co-occurrence of multiple sequence patterns in short genomic regions to discover motifs, which likely decreases false positive predictions compared with methods considering individual patterns separately; and (ii) SIOMICS can identify individually under-represented motifs in addition to overrepresented motifs, because the chance of the occurrence of a combination of multiple motifs in a random sequence is much smaller than that of one individual motif, and underrepresented motifs may be significantly over-represented in input sequences when considered together with their cofactor motifs and thus can be identified as a motif in motif modules. Here a motif module is defined as a group of motifs with binding sites of all motifs in this group co-occurring in significant many short regions; ‘short regions’ refers to genomic regions shorter than 1000 bps. Because of its high accuracy and low time-cost requirement (21), SIOMICS holds great promise for the comprehensive discovery of motifs under a given experimental condition.

We applied SIOMICS (21) to 349 human DHS datasets to predict motifs of potentially all active TFs in each dataset. These datasets were generated by DNase-seq from 349 human samples that could be classified into 30 cell or tissue types (22). We identified 845 to 1325 motifs in each dataset. By representing similar motifs in different datasets with a unique motif, we clustered the predicted motifs in all datasets into 2684 non-redundant motifs. Compared with seven collections of known TF motifs, more than 84.13% of the 2684 predicted motifs were similar to the known motifs, and 54.02 to 75.95% of the known motifs were similar to the 2684 predicted motifs. Because SIOMICS identified motifs through the identification of motif modules, we also predicted 43 663 to 2 013 288 motif modules in each dataset. Compared with eight resources of known interacting TFs, we observed that on average more than 84.23% of the known interacting TFs were represented in our predicted motif modules. We further investigated the genomic locations that the motifs prefer to bind, and found that 45.81% of motifs prefer to bind special types of regions. We also observed that motifs enriched in proximal regions rarely overlapped with motifs enriched in distal regions, motifs enriched in 5′ distal regions were often enriched in 3′ distal regions, etc. Finally, we showed that the predicted motifs reliably defined cell or tissue types of the 349 datasets. All generated resources are freely available at http://server.cs.ucf.edu/predrem/.

## MATERIALS AND METHODS

### DHS data processing

We downloaded 349 DHS datasets from (22) (Supplementary file S1). Each DHS region shorter than 800 bps was extended evenly from its two ends to 800 bps, because the average CRM length is much larger than that of these DHSs (23,24). We also discarded DHSs longer than 5000 bps, because the number of such DHSs was relatively small and including them significantly increased the time cost to predict motifs. We downloaded corresponding DNA sequences for DHSs no longer than 5000 bps from the University of California, Santa Cruz genome browser, with repeats and exon regions masked by ‘N's. The exon regions were defined in the GENCODE version 18 (25).

We assigned the cell or tissue types of the 349 datasets according to the assignment of the same datasets in (26) and known biology for other datasets. We also distinguished fetal tissues from normal tissues. Moreover, we merged the fIntestine_Sm type and the fIntesting_Lg types, because on average 71% of the DHSs in datasets from the two types overlapped (Supplementary file S2). Moreover, 99.17% of the predicted motifs in the two types were similar. In this way, we assigned the 349 datasets to 30 types (Supplementary file S1).

### The pipeline for comprehensive motif discovery

**Figure 1.**
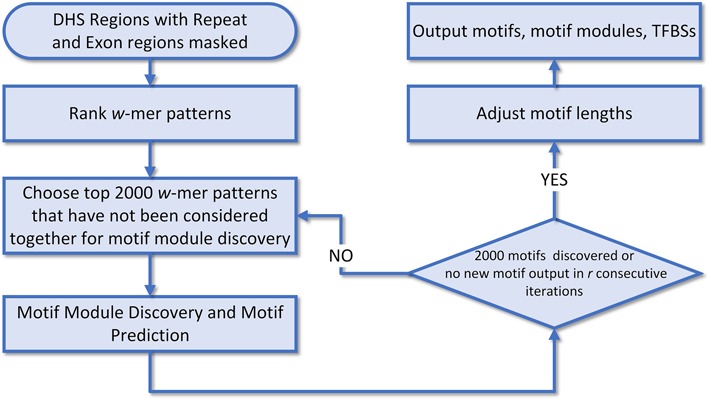
The pipeline to discover motifs in a DHS dataset by SIOMICS.

The pipeline for comprehensive motif discovery by SIOMICS (21) in a DHS dataset was as follows (Figure 1): first, SIOMICS ranked all 8-mer patterns with the repeat-masked sequences from the extended DHS regions (6). SIOMICS used 8-mer patterns as an initial approximation of motifs, because 8-mer patterns already account for the main portion of the majority of TF binding motifs (27–30). Second, SIOMICS took the top 2000 8-mer patterns as candidate motifs to predict motif modules and motifs (6). SIOMICS considered the top 2000 patterns because there are around 1500 sequence-specific-binding TFs in the human genome (2) and one TF may bind multiple motifs (31). The 8-mer patterns contained in predicted motif modules were output as predicted motifs. Third, SIOMICS iteratively ranked the top 2000 patterns and discovered motif modules and motifs, until 2000 motifs were discovered or no new motif was discovered in *r* consecutive iterations (6). Finally, for each obtained motif, SIOMICS extended/shortened the motif based on nucleotides at the neighboring positions of its current TFBSs (Supplementary file S3). Correspondingly, SIOMICS changed the TFBSs of these adjusted motifs and the motif modules composed of these adjusted motifs (21).

### Resources of known motifs and known interacting TF pairs

We downloaded known motifs from seven collections. They were TRANSFAC, JASPAR (2014 Core, non-redundant), HOCOMOCO (v9), FactorBook, a collection from a high throughput study we termed DBS (DNA binding specificity), Neph *et al.* and Kheradpour *et al.* (27–29,32–35). TRANSFAC (28) and JASPAR (29) are two major databases of known motifs. HOCOMOCO contains hand-curated human TF motifs (32). FactorBook stores the human TF motifs identified in ENCODE (33). DBS contains motifs that represent the majority of human TF binding models (34). Neph *et al.* *de novo* identified motifs within ∼50 bps long DNase footprints in 41 DHS datasets (27). Kheradpour *et al.* provided a resource of known and discovered motifs in ENCODE TF-based ChIP-seq datasets (35). We also downloaded the motifs in JASPAR 2010 All Collection as certain motifs such as the FAM (familial binding) motifs and the CNE (conserved non-coding elements) motifs are not included in the JASPAR 2014 Core. We used a compendium of 244 RNA-binding motifs as well, which are identified for RNA-binding proteins from*in vitro* experiments (36).

We extracted known interacting TF pairs from eight resources: BioGRID (Release 3.2.108) (37); DIP (Release 2014/01/17) (38); HPRD (Release 9) (39); IntAct (Release 2014/02/13) (40); MINT (Release 2012/10/29) (41); PIPs (score cut off 0.25) (42); Gerstein *et al.* (43) (http://encodenets.gersteinlab.org/enets1.Proximal_raw.txt); and Ravasi *et al.* (44).

### Enrichment of a motif in a type of regions

We defined 16 types of genomic regions. They were proximal transcription termination site (TSSs), proximal TTSs, 5′ UTRs, 3′UTRs, first introns, other introns, 5 types of 5′ distal regions and 5 types of 3′ distal regions. The proximal TSSs were defined as the regions from the gene start sites of the GENCODE V18 genes to their 2.5 kb upstream (25). The proximal TTSs were the regions from the gene end position of the GENCODE genes to their 2.5 kb downstream. The five types of 5′ and 3′ distal regions were defined with the following five cutoffs: 2.5, 5, 10, 20 or 100 kb. The 5′ >*x* distal regions of a gene were defined as regions that were more than *x* away from the start of this gene and at least *x* away from any other gene, where *x* equal to one of the five cutoffs. Similarly, we defined 3′ >*x* distal regions for the five cutoffs. The 5′ UTRs, 3′ UTRs, first introns and other introns were defined according to GENCODE V18. To analyze motifs shared by proximal TSSs and proximal TTSs, we also defined ‘pure’ proximal TSSs that did not overlap with any proximal TTS, and ‘pure’ proximal TTSs that did not overlap with any proximal TSS. Similarly, we defined ‘pure’ 5′ and 3′ distal regions.

We calculated the enrichment *P*-value of a motif in a type of genomic regions using the binomial test. The parameter of the binomial test was calculated as the ratio of the total number of bps in this type of genomic regions that overlapped with the DHSs in the dataset under consideration to the total number of bps in DHSs in this dataset. If the *P*-value < 0.01 after Bonferroni correction, we considered that the motif was enriched or preferred to occur in this type of genomic regions.

### Cluster motifs from different datasets into non-redundant motifs

For the comparison of predicted motifs with known motifs, we clustered motifs discovered in the 349 datasets. First, we sorted the datasets according to the number of their predicted motifs from the largest to the smallest. Second, we considered every motif from the dataset with the largest number of predicted motifs as an initial non-redundant motif cluster. Third, for the remaining dataset with the largest number of predicted motifs, we determined which motifs in this dataset were similar to the non-redundant motif clusters. A motif x was considered to be similar to a non-redundant motif cluster A, if (i) x was similar to at least one motif in A with the STAMP *E*-value (45) <1E-8; and (ii) x was similar to all other motifs in A with the STAMP *E*-value < 1E-5 when the number of motifs in A was <4, or similar to at least 90% of other motifs in A with the STAMP *E*-value < 1E-5 when the number of motifs in A was >3. If x was similar to A, we added x to the cluster A. For all motifs that were not similar to any cluster, we determined whether these motifs were similar to each other. If multiple motifs were similar, a new cluster containing these motifs was added to the list of the clusters. For every remaining motif, a new cluster with only this motif was added to the cluster list. Fourth, we repeated the third step with each remaining dataset until all motifs in all datasets were considered. We used the motif that was similar to the largest number of other motifs (*E*-value < 1E-5) in the same cluster to represent this cluster. Fifth, we refined clusters by iteratively conducting hierarchical clustering on adjusted motifs contained in clusters with similar representative motifs (Supplementary file S3). Finally, we output the clusters as final non-redundant motifs, with each cluster that contains many similar adjusted motifs represented by one motif.

Alternatively, we started from the dataset with the smallest number of predicted motifs to generate the initial clusters. We then modified these initial clusters by considering motifs from datasets with more and more predicted motifs, using the aforementioned criteria to modify existing clusters and generate new clusters. We also started from GM12878, K562 and H1-hESC, followed by the types of datasets with the largest number of datasets, to cluster motifs. The two alternative procedures generated similar non-redundant motifs and similar numbers of motifs as the first procedure, with the first procedure generating <1% fewer motifs. We thus chose the first procedure for convenience.

## RESULTS

### A brief summary of motif modules and motifs discovered in 349 DHS datasets

We applied SIOMICS (21) to the 349 DHS datasets to discover motifs. On average, we discovered 376 743 motif modules and 1083 motifs in each dataset. The number of motifs in a predicted motif module varied from 2 to 6, with 99.94% of motif modules consisting of 2 to 4 motifs. On average, there were 2.56 motifs in a motif module.

The number of motif modules and motifs discovered in a dataset varied greatly (Supplementary file S1). The five datasets with the most motif modules were all from fetal muscle tissues, with 2 013 288 the largest number of motif modules identified in a dataset. The two datasets with the smallest number of motif modules were from primary T helper cells and contained 43 663 and 48 586 motif modules. The 30 datasets with most predicted motifs were all from fetal tissues with the exception of the dataset 10_CACO2-DS8235, which contained the highest number of adjusted motifs (1325 motifs). Approximately 50% of the 30 datasets with the fewest motifs related to hematopoietic tissues, including five datasets related to human primary T helper cells; the smallest number of adjusted motifs discovered in a dataset was 825.

The number of motif modules discovered in a dataset highly correlated with the number of motifs in the same dataset (Spearman's rank correlation 0.8991). The number of motif modules in a dataset also highly correlated with the average number of motifs in modules in the same dataset (Spearman's rank correlation 0.9358). This average number varied across datasets, with the largest average number from a fetal tissue dataset and the smallest average number from a T helper cell dataset.

The number of datasets where a motif was discovered also differed considerably (Supplementary file S4). To determine in how many datasets a motif occurred, we clustered motifs from all datasets into 2684 clusters and represented each cluster with a unique motif (Supplementary file S4). A total of 14 of the 2684 motifs occurred in all datasets. Another 63 motifs were found in all 30 types of tissues and cells, although not in all datasets. These motifs are likely motifs of house-keeping TFs. For instance, motifs nrMotif1, nrMotif7, nrMotif10 and nrMotif13 were similar to motifs of known house-keeping TFs FOXJ3 (TOMTOM: 0.0131968, STAMP: 7.9533E-7), REST (TOMTOM: 0.499988, STAMP: 1.5034E-8), PRDM4 (TOMTOM: 0.325671, STAMP: 2.6343E-10) and PPARA (TOMTOM: 0.256667, STAMP: 1.4763E-6), respectively (The TOMTOM and STAMP similarity *E*-values were in the parentheses) (45–47). There were also 395 motifs discovered in <5% of the datasets. Many of these 395 motifs were similar to tissue-specific motifs in the literature. For instance, motifs nrMotif2316 and nrMotif2570 were similar to motifs of known tissue-specific TFs HNF1B (TOMTOM: 0.149668, STAMP: 2.1465E-8) and PAX5 (TOMTOM: 0.126543, STAMP: 6.893E-8), respectively (48,49).

Because motifs were often discovered in multiple datasets, the discovered motif modules were also shared by datasets. If motif modules in two datasets were composed of the same subset of the 2684 motifs, we considered them to be shared by the two datasets. We found 9 418 555 shared motif modules in the 349 datasets. The percentage of motif modules shared by two datasets ranged from 58.81 to 99.49%, with an average of 92.0%. Relatively, the percentage of shared modules by datasets from fetal muscle and fetal kidney was not impressively high, whereas the percentages were much higher from tissues such as endothelial, fibroblast, etc (Supplementary file S1).

### The 2684 non-redundant motifs were similar to known motifs in seven large collections

To validate the 2684 motifs, we compared them with motifs collected in seven public repositories (Table 1). We employed two tools, TOMTOM (47) and STAMP (45), to define similar motifs. Based on our experience, STAMP may improperly consider two motifs of very different lengths similar, and TOMTOM cannot distinguish random patterns with low information content from true motifs with high information content (Supplementary file S3). Combining these two tools can address these issues to a large extent. Two motifs were called similar if the TOMTOM comparison *E*-value of the two motifs was <0.5 and the STAMP comparison *E*-value of the two motifs was <1E-4, or the TOMTOM *E*-value was <1 and the STAMP *E*-value was <1E-5.

**Table 1A.** The majority of vertebrate motifs in seven collections were included in our prediction

Collections	#Motifs in the collection	#Motifs in the collection predicted by 2684 nrMotifs	% Motifs in the collection predicted by 2684 nrMotifs	Average #motifs in the collection predicted by 2684 randomly generated motifs	Average% motifs in the collection predicted by 2684 randomly generated motifs
TRANSFAC	522	282	54.02	27.2	5.21
JASPAR	593	328	55.31	36.8	6.21
HOCOMOCO	1896	1210	63.82	101.6	5.36
FactorBook	79	60	75.95	4.3	5.44
DBS	843	458	54.33	52.4	6.22
Neph *et al.*	683	497	72.77	41.5	6.08
Kheradpour *et al.*	2065	1127	54.58	119.8	5.80

We observed that the predicted motifs included the majority of known vertebrate motifs in the seven collections. For instance, 54.02, 55.31 and 63.82% of vertebrate motifs in TRANSFAC, JASPAR 2014 Core and HOCOMOCO, respectively, were similar to the 2684 motifs (Table 1A). Note that SIOMICS does not consider motifs with gaps in the middle, and almost all motifs with no gap identified by Neph *et al.* in ∼50 bps long DHS footprints were similar to our predicted motifs. To assess the significance of the high percentages of known motifs that were similar to the predicted motifs, we generated 2684 random motifs with the same length as the corresponding predicted motifs. Each row of a random motif was constructed by generating four random numbers and then normalized them into numbers between 0 and 1 so that the sum of the normalized four numbers was 1. We compared these random motifs with the motifs in the seven repositories in the same way. We repeated this procedure 10 times, and found that, on average, <6.27% of the known motifs in these collections were similar to the 2684 random motifs (Table 1A).

We further focused on the predicted motifs in GM12878 and K562, as ENCODE generated more TF-based ChIP-seq data in these two cell lines. The ENCODE project predicted 36 motifs in GM12878 and 47 motifs in K562 and stored them in FactorBook (33), by predicting five motifs in the 100-bps regions around the summits of the top 500 peaks in each TF-based ChIP-seq dataset. We found that 20 out of 36 motifs in GM12878 and 33 out of 47 motifs in K562 were similar to the predicted motifs in the two cell lines (Table 1B). The missed FactorBook motifs were likely because the motifs represented by TFBSs in the top 500 peaks were slightly different from those from all peaks. Since ENCODE used only the top 500 peaks, we applied a popular tool DREME (14) to all peaks of the same ChIP-seq datasets to identify five motifs in each dataset. DREME predicted 195 and 259 motifs in the two cell lines (Supplementary file S5). We found that about 76.41 and 80.69% of the top motifs predicted by DREME on TF-based ChIP-seq datasets were similar to our predicted motifs in DHS datasets of the corresponding cells (Table 1B). We also compared the predicted motifs with the motifs identified by Kheradpour *et al.* in these ChIP-seq datasets (35). We found that 64.18 and 67.19% of the motifs identified by Kheradpour *et al.* (35) were similar to our predicted motifs in the corresponding cells (Table 1B). Because peak regions from individual TF-based ChIP-seq experiments were often under-represented in DHSs from a DNase-seq experiment under the same condition, the above comparisons suggested that SIOMICS can identify under-represented motifs with high accuracy. It also implied that the majority of motifs identified directly from TF-based ChIP-seq experiments were included in our prediction based on DHSs under the same conditions.

**Table 1B.** Known motifs in GM12878 and K562 were included in our prediction

	#Motifs predicted	Collection	#Motifs in the collection	#Motifs in the collection predicted	%Motifs in the collection predicted
		factorbook_gm12878	36	20	55.56
GM12878	961	dreme_gm12878	195	149	76.41
		Kheradpour *et al.*_gm12878	67	43	64.18
		factorbook_k562	47	33	70.21
K562	953	dreme_k562	259	209	80.69
		Kheradpour *et al.*_k562	64	43	67.19

It is also worth mentioning that most predicted motifs were also similar to known motifs (Supplementary file S4). We observed that 2258 (84.13%) of the 2684 predicted motifs were similar to at least one motif in the seven motif collections. We considered these 2258 predicted motifs as known motifs. A total of 379 of the 426 remaining motifs were similar to at least one motif in the seven collections together with the JASPAR 2010 All Collection with TOMTOM *E*-value < 1 or STAMP *E*-value < 1E-4. Only 47 predicted motifs were likely new motifs that were not similar to any motif with TOMTOM *E*-value < 1 or STAMP *E*-value < 1E-4. Such a high percentage (>84.13%) of motifs similar to known motifs indicated that the predicted motifs were likely biologically meaningful.

To evaluate whether the predicted motifs may also represent motifs other than TF binding DNA motifs, we compared the predicted motifs with the 244 RNA-binding motifs described in (36). We found that 65.98% of RNA motifs in (36) were similar to the 2684 motifs. Moreover, 654 (24.37%) out of the 2684 predicted motifs were similar to at least one RNA motif.

### The 47 new motifs are likely functional

To investigate the functionality of the 47 new motifs, we studied the functions of their cofactors in the predicted motif modules. For every new motif, we collected all predicted motif modules containing at least two known motifs and this new motif. We then examined whether the known motifs in a motif module shared functions described by similar gene ontology (GO) terms using GOTermFinder (50). If yes, we checked whether target genes of the motif modules significantly shared similar GO terms. Here the target genes of a motif module were approximated as the genes closest to the DHSs containing TFBSs of the motif module. We found that for 43 out of the 47 new motifs, their cofactors shared similar functions and such shared functions were also shared by their target genes, which implied that the new motifs may have similar functions to those of their cofactors.

For instance, in the dataset 117_fKidney_renal_pelvis-DS20448, the adjusted motif M699 was found in multiple motif modules with three known cofactor motifs, TCF12, TFAP2C and SMARCB1. TCF12, TFAP2C and SMARCB1 share several GO terms related to positive regulation of metabolic process, such as GO:0010604 (positive regulation of macromolecule metabolic process) and GO:0051173 (positive regulation of nitrogen compound metabolic process). Consistently, the target genes of the motif modules formed by M699 and the motifs of the three TFs significantly shared the functions GO:0010604 (multiple comparison corrected *P*-value 6.71E-04) and GO:0051173 (multiple comparison corrected *P*-value 1.03E-05) (50). Such a consistency of functions of cofactors and target genes supported the functionality of the motif modules in metabolic regulation. It also strongly implied that the new motif was functional and possessed similar functions to those of the cofactors.

### TFBSs of 45.81% motifs prefer to occur in specific genomic regions

**Figure 2.**
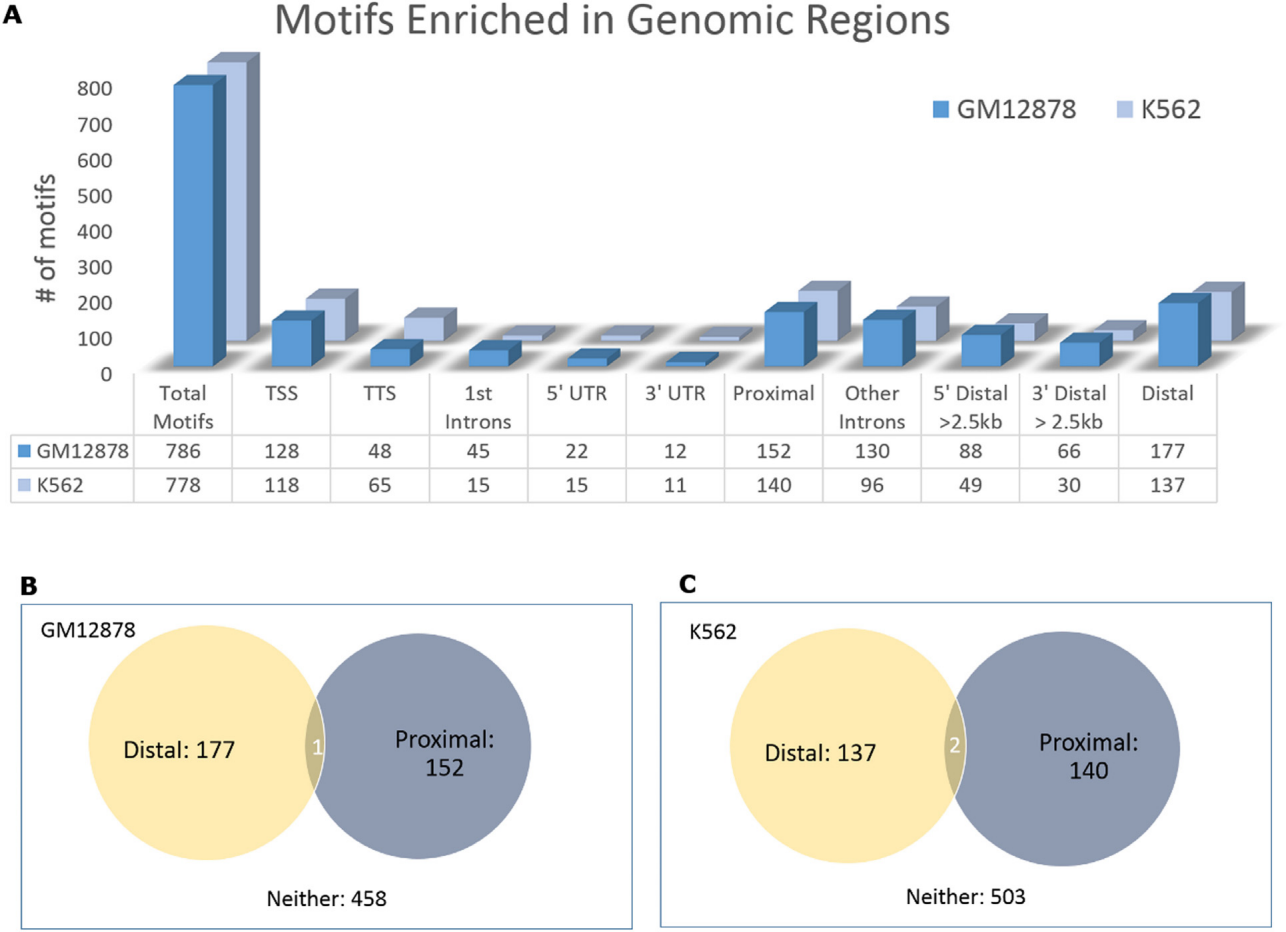
(**A**) The number of motifs enriched in different types of regions. (**B**) The overlap of motifs enriched in distal and proximal regions in GM12878. (**C**) The overlap of motifs enriched in distal and proximal regions in K562.

To see which type of genomic regions the predicted motifs prefer to bind, we compiled 16 types of genomic regions and calculated the enrichment *P*-value of every motif in each type of regions. We found that on average, TFBSs of 45.81% of motifs in a dataset preferred to occur in specific types of genomic regions (Figure 2A).

Figure 2A showed the number of motifs enriched in different types of regions in two representative datasets, GM12878 and K562. We observed that motifs enriched in proximal regions (proximal TSSs, proximal TTSs, 3′ UTRs, 5′UTRs and first introns) were rarely enriched in distal regions (all types of 5′ and 3′ distal regions and other introns). For instance, 152 and 177 motifs were enriched in proximal regions and distal regions in GM12878, respectively, whereas only one motif was enriched in both types of regions (Figure 2B). Similarly, this was true for K562 (Figure 2C) and other datasets.

We also noticed that motifs enriched in 5′ distal regions often preferred to bind regions more distant to TSSs. Similarly, motifs enriched in the 3′ distal regions often preferred to bind regions more distant to TTSs. For example, in GM12878, 88 motifs were enriched in 5′ >2.5 kb distal regions, 78, 68 and 65 of which were enriched in 5′ >5, >10 and >20 kb distal regions, respectively. Similarly, in K562, 49 motifs were enriched in 5′ >2.5 kb distal regions, 43, 41 and 36 of which were enriched in 5′ >5, >10 and >20 kb distal regions, respectively.

Genomic region enrichment analysis also revealed other new features of motifs: motifs enriched in proximal TTSs were often enriched in proximal TSSs, and motifs enriched in 3′ distal regions were often enriched in 5′ distal regions. In fact, for all 349 datasets, on average, 87.60% of proximal TTS enriched motifs were also proximal TSS enriched (maximum 97.67%, minimum 57.14%). Although in general fewer motifs were enriched in proximal TTSs than those in proximal TSSs, an average of 58.89% of proximal TSS enriched motifs were still proximal TTS enriched. Moreover, on average, 64.53% of 5′ distal enriched motifs were also 3′ distal enriched, and 80.38% of 3′ distal enriched motifs were also 5′ distal enriched (Supplementary file S6). Because proximal TTS regions may overlap with proximal TSS regions and 3′ distal regions may overlap with 5′ distal regions, we further generated a list of ‘pure’ proximal TSS, proximal TTS, 5′ distal and 3′ distal regions (‘Materials and Methods’ section), and repeated the same enrichment analyses. We found that on average, 58.81% of ‘pure’ proximal TTS enriched motifs were still ‘pure’ proximal TSS enriched, and 38.25% of ‘pure’ 3′ distal enrichment motifs were ‘pure’ 5′ distal enriched. Note that the dramatic decrease of the shared enriched motifs by ‘pure’ 3′ distal regions and ‘pure’ 5′ distal regions was caused by the removal of middle regions between all pairs of adjacent genes on the strands, which prevents from the consideration of many shared enriched motifs. Despite the percentage decrease of shared motifs (58.81 versus 87.60% for ‘pure’ proximal TTS enriched motifs and 38.25 versus 80.38% for ‘pure’ 3′ distal motifs), a large percent of ‘pure’ proximal TTS enriched motifs are still ‘pure’ proximal TSS enriched, and a large fraction of motifs enriched in ‘pure’ 3′ distal regions are still enriched in ‘pure’ 5′ distal regions.

The predicted genomic region enrichment of motifs was also supported by the literature. For instance, typical core promoter TFs such as E2F4, E2F6, MAX, SP1 and SP2 were shown to have significant proximal promoter bias (43), which agreed with our predictions in GM12878 and K562. The predicted cell-specific TFBS enrichment of HNF4A in HepG2 but not in GM12878, K562, HeLa and H1-hESC, perfectly agreed with a recent study (51).

### The predicted motif modules contain motifs of most known interacting TFs

To validate the predicted motif modules, we compared TF pairs corresponding to the predicted motif pairs in motif modules with known interacting TF pairs from eight resources (Table 2). The TF corresponding to a predicted motif was determined as the TF whose motifs in HOCOMOCO were most similar to this predicted motif (32). We found that on average, 84.23% of known interacting TF pairs in each resource were included in our predictions (Table 2). Such a high percentage strongly supported the near-comprehensiveness of our predictions. It also demonstrated the good accuracy of SIOMICS and the functionality of the predicted motif modules.

**Table 2.** Most known interacting TF pairs were included in our predictions

Resources	#Interactions in the resource	#Interactions in the resource related to TFs in our study	# Known interactions discovered	%Known interactions discovered
BioGRID	155 100	2769	2357	85.12%
DIP	3060	90	87	96.67%
HPRD	39 184	1397	1174	84.04%
IntAct	267 000	931	673	72.29%
MINT	25 756	198	174	87.88%
PIPs	78 613	1265	1027	81.19%
Gerstein, M. B. *et al.*	519 691	13 211	10 876	82.33%
Ravasi, T. *et al.*	5238	1278	1078	84.35%

We also found that between 12.55 and 24.40% of the predicted motif pairs in a dataset were supported by known interactions (Supplementary file S7). For example, 1336 (15.57%) of the 7196 potential TF–TF interaction pairs discovered in GM12878 were verified by known interactions. The percentages of the predicted modules validated are likely under-estimated, due to the limited number of experimentally confirmed TF–TF interactions available (Table 2). It is also possible that co-binding events identified in a portion of the predicted modules may not represent interactions between TFs. For instance, in a motif module composed of motifs of three TFs, two TFs may not interact with each other while both interact with the third one.

### The discovered motifs accurately classify different types of DHS datasets

We investigated whether the 2684 non-redundant motifs could predict the cell and tissue types of the DHS datasets. The 349 datasets were grouped into 30 types (Supplementary file S1), among which 16 types had seven or fewer datasets (eight types each had only one dataset). We excluded the types with seven or fewer datasets, as their sample sizes may be too small to reliably classify datasets (26). In this way, we obtained 310 datasets from 14 cell or tissue types to be classified (Supplementary file S1).

**Figure 3.**
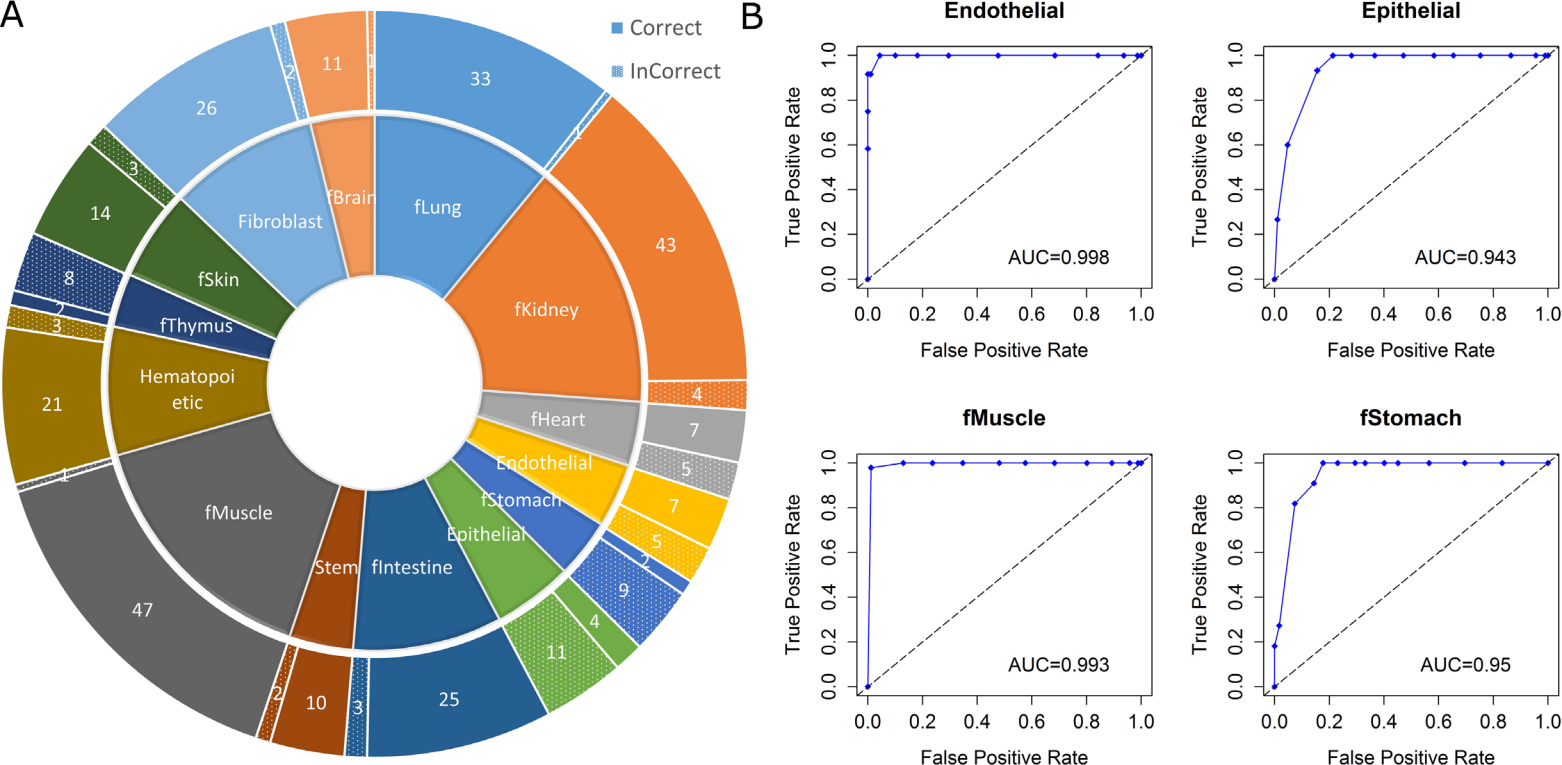
(**A**) Classification of 310 datasets using 2684 motifs. (**B**) Individual ROC curves for four tissue types.

We built a support vector machines (SVM) classification model using the sequential minimal optimization (SMO) algorithm (52,53) to classify the 310 datasets using 10-fold cross validation. In total, 252 (81.29%) of the 310 datasets were correctly classified (Figure 3A). The highest, lowest and median accuracy in the 10-fold validation was 93.55, 67.74 and 80.65%, respectively. We further performed the receiver operating characteristic (ROC) analysis of these 14 classes by pairwise comparison. We compared each class with all other classes (54). The area-under-curve (AUC) scores for the 14 classes ranged from 0.943 to 1, with a weighted AUC score of 0.973 (Figure 3B, Supplementary file S8). For the 58 ‘misclassified’ datasets, six from fThymus were predicted as Hematopoietic. This may be explained by the so-called ‘extramedullary haematopoiesis’, which shows that the liver, thymus and spleen may resume their haematopoietic function if necessary (55). Moreover, nine datasets from epithelial were classified as fibroblast. This misclassification may be explained by the process called epithelial-mesenchymal transition, in which epithelial cells can give rise to fibroblasts (56). In addition, 24 datasets from different fetal tissues were classified as other types of fetal tissues. For instance, six datasets from ‘fStomach’ were misclassified as ‘fKidney’. This type of misclassification of different fetal tissues might be caused by the biological similarity of different fetal tissue types. The remaining 19 ‘misclassified’ datasets may represent the classification errors or the unknown similarities of the cell or tissue types.

## DISCUSSION

We applied SIOMICS (21) to discover DNA motifs in 349 DHS datasets. On average, we identified 1083 motifs and 376 743 motif modules in one dataset. The identified motifs were clustered into 2684 non-redundant motifs. Compared with known motifs, more than 84.13% of the predicted motifs were similar to known motifs. Conversely, 54.02 to 75.95% of known motifs were similar to the predicted motifs. Compared with known TF–TF interactions, more than 12.55% of predicted motif pairs in motif modules corresponded to the motif pairs of known interacting TF pairs, and on average 84.23% of known interacting TF motifs were represented in the predicted motif modules. We also showed that the predicted motifs reliably classified different types of DHS datasets. Our predictions demonstrated the powerfulness of the SIOMICS method and provided valuable information for future studies on gene transcriptional regulation in these cells and tissues.

Approximately 1500 sequence-specific binding TFs are known in the human genome while we predicted 2684 motifs. This paradox may be partially explained by the following three factors. First, certain TFs bind motifs of different forms (4,31). For instance, two motifs, nrMotif677 and nrMotif2540, were similar to different HSF1 motifs in HOCOMOCO (HSF1_f1 and HSF1_do with STAMP *E*-values 1.0266E-9 and 2.61E-07, respectively). However, the two predicted motifs were completely different from each other and thus counted as two different motifs. Second, the clustering procedure we applied is imperfect. The problem of clustering similar motifs with complete linkage is an NP-hard problem, because this problem is similar to the NP-hard problem of identifying all cliques in a graph. In other word, we had to take a polynomial time procedure in clustering, which affected the resulted 2684 motifs. Third, motifs other than the TF binding motifs exist in DHSs, as 65.98% of known RNA motifs were shown to be similar to our predicted motifs.

Even with the comprehensive prediction of motifs, several issues may still prevent from accurate TFBS prediction in DHSs. These issues include the largely unknown competition and cooperation of TFs and other regulatory proteins, the elusive features of weak TFBSs that may depend on both TF concentration and others, etc. Therefore, although the motifs and motif modules predicted in this study are likely highly reliable, the predicted TFBSs still need significant refinement. For instance, SIOMICS predicted only 31.90% of CTCF (CCCTC-binding factor) TFBSs and 18.56% of EGR-1 (Early growth response protein 1) TFBSs in K562, compared with the prediction of 44.74% of CTCF TFBSs and 12.16% of EGR1 TFBSs in K562 by centipede (57), a popular method for TFBS discovery (Supplementary file S9).

The time and space cost of predicting motifs by SIOMICS on the 349 DHS datasets was relatively low. In a dataset of an average size, there were 169 744 sequences of 903 bps long. The average running time on one dataset by SIOMICS was 93 CPU hours on a compute core of Intel Xeon 64-bit processor (Quad Core at 3.0 GHz and 4GB RAM for each core). We neglected DHS regions longer than 5000 bps in our analyses, not because SIOMICS could not handle these long sequences, but because these long sequences significantly increased the SIOMICS running time.

We also built an SVM classification model using the SMO algorithm to classify the 341 datasets into 22 types using 10-fold cross validation (the eight types with only one dataset excluded). We found that 248 (72.73%) of the 341 datasets were correctly classified. For the misclassified datasets, we found that all types with a small number of datasets (<8) were misclassified. For instance, four out of four Keratinocyte, three out of three fSpinal_cord and four out of four fPlacenta were misclassified. We thus discarded these types of datasets and analyzed the remaining 310 datasets.

Although we strived to discover motifs of all active TFs in each dataset, we may miss certain motifs (Supplementary file S10). To discover even more motifs in a dataset, we could have required more than 2000 top 8-mer patterns be considered for motif module discovery. In fact, we identified 2000 motif candidates in most datasets before adjusting motif lengths, which implied that we could have considered more top 8-mer patterns. However, we only considered the top 2000 8-mer patterns in this study, because we wanted to run SIOMICS with the same parameters on all datasets.

We provided a useful resource at http://server.cs.ucf.edu/predrem/. This resource contains motifs, motif modules and TFBSs predicted in 349 datasets from 30 different tissue or cell types. This resource will be an important addition to the current repositories of TFs and their interactions. It will also serve as an excellent starting point for tissue-specific gene transcriptional regulation studies.

## SUPPLEMENTARY DATA

Supplementary Data are available at NAR Online.

SUPPLEMENTARY DATA
