# Supplementary material for: Comprehensive discovery of DNA motifs in 349 human cells and tissues reveals new features of motifs
Source: Nucleic Acids Res. 2014 Dec 10;43(1):74–83. doi: 10.1093/nar/gku1261 (PMC4288161; doi:10.1093/nar/gku1261)
Supplement: SUPPLEMENTARY DATA [file supp_43_1_74__index.html]

Comprehensive discovery of DNA motifs in 349 human cells and tissues reveals new features of motifs — SUPPLEMENTARY DATA 

# Comprehensive discovery of DNA motifs in 349 human cells and tissues reveals new features of motifs

## SUPPLEMENTARY DATA

**Files in this Data Supplement:**

- SUPPLEMENTARY DATA
- SUPPLEMENTARY DATA
- SUPPLEMENTARY DATA
- SUPPLEMENTARY DATA
- SUPPLEMENTARY DATA
- SUPPLEMENTARY DATA
- SUPPLEMENTARY DATA
- SUPPLEMENTARY DATA
- SUPPLEMENTARY DATA
- SUPPLEMENTARY DATA
