# Supplementary material for: Comprehensive discovery of DNA motifs in 349 human cells and tissues reveals new features of motifs
Source: Nucleic Acids Res. 2014 Dec 10;43(1):74–83. doi: 10.1093/nar/gku1261 (PMC4288161; doi:10.1093/nar/gku1261)
Supplement: SUPPLEMENTARY DATA [file supp_gku1261_Supplementary_File_S3.docx]

**The extended SIOMICS method**

The original SIOMICS ([1](#_ENREF_1)) starts by ranking all *w*-mer (default *w*=8) patterns in input sequences. It then iteratively chooses top *m* *w*-mer patterns and predicts motifs. The top *m w*-mer patterns used every time consist of the predicted motifs from previous iterations together with those top *w*-mer patterns that have not been considered simultaneously with these predicted motifs so far. The prediction of motifs every time is through the prediction of motif modules, in which every group of statistically significant co-occurring *w*-mer patterns is output as a motif module, with all *w*-mer patterns in predicted motif modules as predicted motifs. The co-occurring instances of the *w*-mer patterns of a motif module in input sequences are defined as the TFBSs of the motifs in this motif module. The iteration is repeated until *m* motifs are discovered or no new motif is found in *r* consecutive iterations. Here, *m* and *r* are parameters input by users. At the end, there will be no more than *m* predicted motifs, each of which is *w* bps long (default *w*=8).

The extended SIOMICS ([2](#_ENREF_2)) starts from the predicted motifs by the original SIOMICS ([1](#_ENREF_1)) to output motifs of different lengths. For simplicity, the extended SIOMICS is still called SIOMICS below. For each discovered motif by the above procedure, SIOMICS collects all its predicted TFBSs in input sequences. SIOMICS then considers the neighboring positions around the predicted TFBSs to determine whether this motif can be extended. The intuition behind this approach is that if this motif is longer than *w*, it is expected that the neighboring positions around most predicted TFBSs likely share a common nucleotide, on the left side, on the right side, or on both sides. SIOMICS applies a binomial test to assess whether the neighboring positions significantly share the same nucleotide(s). If the binomial p-value is smaller than 1E-10, SIOMICS extends the motif length by 1. SIOMICS extends the motif one position at a time, from the left side to the right side of the currently predicted TFBSs. When no extension can be made, SIOMICS output the final form of the motif. If no extension is made from the beginning on either side, SIOMICS also considers extending two adjacent positions at a time and repeats the above process until no more extension can be made.

There may be motifs unchanged after the above extension step. For these motifs, SIOMICS considers reducing their lengths. SIOMICS compares these unchanged motifs to see whether two motifs are similar. Two motifs *a* and *b* are similar, if *a* and *b* match each other at *w-1* positions, or share a substring of *w-1* bps long. Moreover, SIOMICS checks whether these similar motifs form motif modules with a common group of other motifs. If both criteria are satisfied, SIOMICS merges these similar motifs and represent them with their shared portions. With these adjusted (extended and shorten) motifs, we run SIOMICS one more iteration to output the final motif modules and their TFBSs.

**The Refined Clustering Method**

Based on the first four steps described in the main text, we have generated 3894 motif clusters (non-redundant motifs) that contain every adjusted motif predicted in the 349 datasets. Each cluster is represented by a motif, which belongs to this cluster and is similar to the largest number of others motifs in the same cluster (E-value < 1E-5). We found that among these representative non-redundant motifs, some of them are still similar to each other. We thus iteratively conduct hierarchical cluster on similar motif clusters (E-value < 1E-5) based on the following steps.

In each iteration, every cluster is considered once and only once. Each time, we choose one cluster from the set of clusters that have not been considered in this iteration until all clusters are considered. For a chosen cluster, we find other clusters that are similar to this cluster (representative motifs of the two clusters have the STAMP similarity E-value < 1E-5. We do not consider the TOMTOM similarity E-value here as the predicted adjusted motifs in general have similar motif lengths instead of dramatically different motif lengths). We then put all adjusted motifs in these similar clusters together to conduct hierarchical clustering with the centroid Linkage. New clusters are formed by cutting the dendrogram where the STAMP similarity E-value is larger than 1E-5. Clusters with more than two motifs are considered as new clusters and used in the next iteration, while clusters with only one or two motifs are considered as outliers, and are put aside. It is also worth mentioning that in the 1st iteration, before conducting clustering, we merged adjusted motifs whose consensus are exactly the same or very similar to the consensus of the representative motif of the cluster under consideration. The criteria of adjusted motif similar to the representative motifs are defined as a) any adjusted motif, whose length is smaller than the length of the representative motif, and its consensus is completely included in the representative motif’s consensus; or b) any adjusted motif, whose length is larger than or equal to the length of the representative motif, and its consensus contains any N-1 sub-sequence of the consensus of the representative motif, where N is the length of the consensus of the representative motif. We also require that the length difference between the adjusted motif and the representative motif is no more than 2 before merging. Merging these similar motifs not only makes sure similar motifs are put in the same cluster, but also greatly reduced the number of motifs to be clustered. We stop the iteration when the number of clusters does not decrease anymore.

The above iteration re-clusters adjusted motifs in clusters with their representative motifs very similar (E-value < 1E-08). In this process, there are outlier clusters generated, which contain one or two adjusted motifs. After the above iteration stops, we assigned all outliers generated in the all previous iterations into the final clusters based on their similarity with the representative motifs. That is, each outlier is assigned to the cluster whose representative motifs are most similar to this outlier motif.

**Criteria of Motif Validation**

A combination of STAMP and TOMTOM, two of the most popular motif similarity comparison tools, is used for the validation with known motifs. This is because based on our experience, STAMP may consider two motifs with very different lengths similar to each other, and TOMTOM cannot distinguish random patterns with low information content from true motifs with high information content. For instance, when comparing the motif A-box in FactorBook with our predicted motif nrMotifs1069, STAMP returned an E-value of 4.5700e-06, while TOMTOM returned an E-value of 2.34226. The two motifs are quite different while STAMP considers them similar motifs.

The alignment of these two motifs is

TAACCACTGAGCCACCGAGCC (FactorBook A-box)

TAACCACA (nrMotif1069, reverse complement)
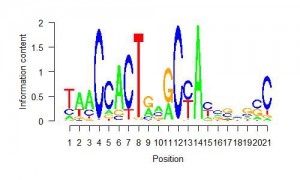

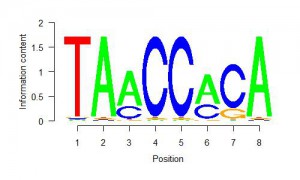


Another example is when comparing a randomly generated motif with known motifs in JASPAR2014 Core, the first match returned by TOMTOM is MA0048 with E-value 0.393205 (See the following figure), while STAMP returned no significant motif match. Each row of this random motif is generated by generating four random numbers independently, and normalizing these numbers into numbers between 0 and 1 so that their sum is 1. This random motif has an information content of 3.82 while its similar JASPAR 2014 Core motif found by TOMTOM has an information content of 14.13. Here when we calculate the information content, we use 0.25, 0.25, 0.25, 0.25 as the frequency of A, C, G, T in the background sequences.


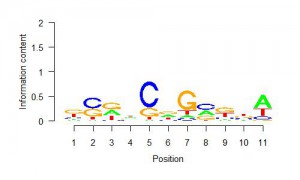

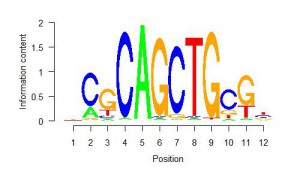


We consider two motifs are similar, if their STAMP comparison E-value < 1E-5 and TOMTOM comparison E-value <1, or their STAMP comparison E-value < 1E-4 and TOMTOM comparison E-value <0.5. The STAMP E-value cutoff 1E-5 and the TOMTOM E-value cutoff of 0.5 are used in multiple studies previously ([3-7](#_ENREF_3)).

1. Ding, J., Hu, H. and Li, X. (2014) SIOMICS: a novel approach for systematic identification of motifs in ChIP-seq data. *Nucleic acids research*, **42**, e35.

2. Ding, J., Dhillon, V., Li, X. and Hu, H. (2014) Systematic Discovery of Cofactor Motifs from ChIP-seq Data by SIOMICS. *Methods*, Accepted.

3. Guo, Y., Mahony, S. and Gifford, D.K. (2012) High resolution genome wide binding event finding and motif discovery reveals transcription factor spatial binding constraints. *PLoS computational biology*, **8**, e1002638.

4. Hu, J., Wang, D., Li, J., Jing, G., Ning, K. and Xu, J. (2014) Genome-wide identification of transcription factors and transcription-factor binding sites in oleaginous microalgae Nannochloropsis. *Scientific reports*, **4**.

5. Dubos, C., Kelemen, Z., Sebastian, A., Bülow, L., Huep, G., Xu, W., Grain, D., Salsac, F., Brousse, C. and Lepiniec, L. (2014) Integrating bioinformatic resources to predict transcription factors interacting with cis-sequences conserved in co-regulated genes. *BMC genomics*, **15**, 317.

6. Seidl, M.F., Wang, R.-P., Van den Ackerveken, G., Govers, F. and Snel, B. (2012) Bioinformatic inference of specific and general transcription factor binding sites in the plant pathogen Phytophthora infestans. *PloS one*, **7**, e51295.

7. Hussong, M., Börno, S., Kerick, M., Wunderlich, A., Franz, A., Sültmann, H., Timmermann, B., Lehrach, H., Hirsch-Kauffmann, M. and Schweiger, M. (2014) The bromodomain protein BRD4 regulates the KEAP1/NRF2-dependent oxidative stress response. *Cell death & disease*, **5**, e1195.
