# Supplementary material for: Comprehensive discovery of DNA motifs in 349 human cells and tissues reveals new features of motifs
Source: Nucleic Acids Res. 2014 Dec 10;43(1):74–83. doi: 10.1093/nar/gku1261 (PMC4288161; doi:10.1093/nar/gku1261)
Supplement: SUPPLEMENTARY DATA [file supp_gku1261_Supplementary_File_S8.docx]

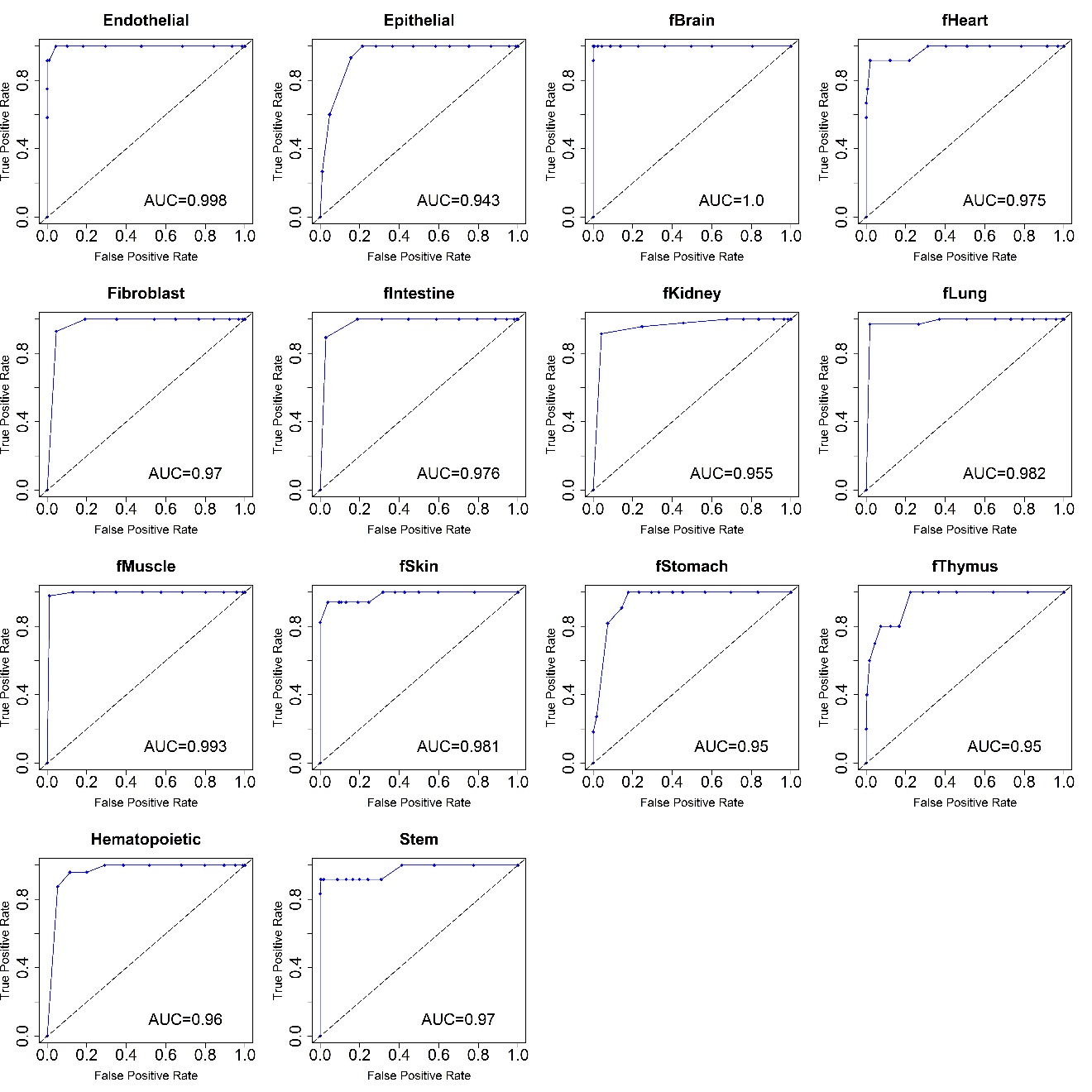


Supplementary Figure S1. Individual ROC curves for 14 tissue types in the classification of 310 datasets using 2684 motifs
